# Long-Term Mechanical Ventilation in Neonates: A 10-Year Overview and Predictive Model

**DOI:** 10.3389/fped.2021.689190

**Published:** 2021-07-13

**Authors:** Michaël Sauthier, Nicolas Sauthier, Krystale Bergeron Gallant, Gregory A. Lodygensky, Atsushi Kawaguchi, Guillaume Emeriaud, Philippe Jouvet

**Affiliations:** ^1^Research Center of Sainte-Justine Hospital, Centre Hospitalier Universitaire Sainte-Justine, Université de Montréal, Montréal, QC, Canada; ^2^Department of Pediatrics, Centre Hospitalier Universitaire Sainte-Justine, Université de Montréal, Montréal, QC, Canada; ^3^Department of Anesthesia, Centre Hospitalier de l'Université de Montréal, Université de Montréal, Montréal, QC, Canada; ^4^Department of Intensive Care Medicine, Pediatric Critical Care Medicine, Tokyo Women's Medical University, Tokyo, Japan

**Keywords:** children, machine learning, prolonged mechanical ventilation, clinical decision support, hospital mortality, critical care

## Abstract

**Objectives:** Significant resources are devoted to neonatal prolonged mechanical ventilation (NPMV), but little is known about the outcomes in those children. Our primary objective was to describe the NPMV respiratory, digestive, and neurological outcomes at 18 months corrected age. Our second objective was on the early identification of which patients, among the NPMV cohort, will need to be ventilated for ≥125 days, which corresponded to the 75th percentile in the preliminary data, and to describe that subgroup.

**Methods:** In this retrospective cohort study, we included all children born between 2004 and 2013 who had a NPMV (≥21 days of invasive or noninvasive respiratory support reached between 40 and 44 weeks of postconceptional age). We used random forests, logistic regression with penalization, naive Bayes, and XGBoost to predict which patients will need ≥125 days of ventilation. We used a Monte Carlo cross validation.

**Results:** We included 164 patients. Of which, 40% (*n* = 66) were female, and the median gestational age was 29 weeks [interquartile range (IQR): 26–36 weeks] with a bimodal distribution. Median ventilation days were 104 (IQR: 66–139 days). The most frequently associated diagnoses were pulmonary hypertension (43%), early pulmonary dysplasia (41%), and lobar emphysema (37%). At 18 months corrected age, 29% (*n* = 47) had died, 59% (*n* = 97) were free of any respiratory support, and 45% (*n* = 74) were exclusively orally fed. A moderate area under the ROC curve of 0.65 (95% CI: 0.54–0.72) for identifying patients in need of ≥125 days of ventilation at inclusion was achieved by random forests classifiers. Among the 26 measured at inclusion, the most contributive ones were PCO_2_, inspired O_2_ concentration, and gestational age. At 18 months corrected age, patients ventilated for ≥125 days had a lower respiratory weaning success (76 vs. 87%, *P* = 0.05), lower exclusive oral feeding proportion (51 vs. 84%, P < 0.001), and a higher neurological impairment (median Pediatric Cerebral Performance Category score 3 vs. 2, *P* = 0.008) than patients ventilated for < 125 days.

**Conclusion:** NPMV is a severe condition with a high risk of mortality, neurological impairment, and oral feed delay at 18 months. Most survivors are weaned of any respiratory support. We identified the risk factors that allow for the early identification of the most at-risk children of long-term ventilation with a moderate discrimination.

## Introduction

Neonates requiring prolonged mechanical ventilation require significant resources and are at a high risk of multiple and serious long-term complications (1). The exact incidence of neonatal prolonged mechanical ventilation (NPMV) is unknown, but around 3% of ventilated children are supported for more than 21 days (2–4). Despite that this group represents a minority of patients, it is responsible for the majority of the economic burden and is increasing in the last decade (4–6). Except for isolated prematurity where the risk factors and outcomes of bronchopulmonary dysplasia have been well-described (7–11), infants with congenital anomalies that cannot be discharged through a home ventilation program have been less described in the recent literature (12). In 2017, our team found the most widely used pediatric definition for prolonged mechanical ventilation that included all children who were still ventilated and who had at least 21 consecutive days of ventilation after 37 weeks postmenstrual age (4). The main advantage of this definition is its applicability to a broad population of newborns (preterm, congenital anomalies, infections, etc.). Those patients are at a high-risk of impaired development (13, 14). Earlier multidisciplinary interventions aim to improve long-term outcomes, but no data or tools are available to help the clinician identify the most at-risk patients.

Our primary objective of this study was to describe the respiratory, neurological, and digestive functional status of the NPMV population at 18-months corrected age, an important milestone in children's follow-up in our institution. Our second objective was to build a predictive model for the early identification of which patients, belonging to more than the 75th percentile, will be ventilated, and to describe the outcomes of this subgroup.

## Materials and Methods

We included all neonates born between April 2004 and December 2013 and admitted in the NICU (65-bed level III unit) of Sainte-Justine Hospital (Montreal, Canada), with a NPMV diagnosis. Using our proposition of pediatric prolonged mechanical ventilation (4), NPMV was defined as at least 21 consecutive days of any ventilatory support (invasive, noninvasive, and high-flow nasal cannula) reached between 40- and 44-weeks postmenstrual age. The ventilatory support had to be more than just a nocturnal application (i.e., ≥6 h a day) to be counted. A ventilation episode was considered continuous if the interruption in mechanical ventilation support was <48 h. Patients with a neurologic death diagnosis or transferred to another institution before inclusion were excluded. The institutional review board approved this retrospective cohort study and waived the need for individual consent (reference number 3872).

### Data Collection

The charts were reviewed by MS and KBG. Data collection quality process was validated on the first 10 patients who were reviewed by both MS and KBG to standardize data collection between both researchers. The charts were reviewed for demographic data, primary and secondary diagnoses, perinatal history in the child and mother's chart, and all ventilatory episodes. Data were manually collected and validated from paper charts into an electronic case report form (eCRF). The eCRF had alerts for common typographical errors or impossible physiological values. It also automatically calculated the observation times (inclusion, 18 months corrected age) based on the date of birth and gestational age. Data were collected at inclusion (when the NPMV diagnosis was first met) and at 18 months corrected age (±1 month). Individual birth weight *Z*-scores were automatically calculated using Olsen's growth curves (15) and inclusion, and 18-month weight *Z*-scores used the World Health Organization growth curve using the corrected age (16, 17). Neurologic impairment was estimated at 18 months corrected age using the Pediatric Overall Performance Category (POPC) and Pediatric Cerebral Performance Category (PCPC) scores (18). These scores are well-validated for the functional neurological status in the pediatric critical care population. Although they can only provide a global assessment, they can be done retrospectively using the patient's chart.

### Statistical Analysis and Missing Values

We described the patient population using median and interquartile range (IQR) for continuous variables and count with percentages for categorical variables and mortality. Considering the small sample size, we conducted the analyses using the Fisher's exact test for nominal variables and the Mann–Whitney *U*-test for continuous variables. We used a linear regression to estimate the slope of the number of patients per year. We considered *P* < 0.05 to be significant. We performed multivariate logistic regressions adjusted for gestational age and gender and reported the adjusted odds ratio (aOR). The statistical analyses were conducted in R 4.0.3 (R Project for Statistical Computing, RRID:SCR_001905) using the tidyverse package (tidyverse, RRID:SCR_019186).

Missing values were limited by the alerts provided by the eCRF. If the information remained missing, these values were inferred if possible (e.g., ventilation mode based on the other available parameters). If missing values remain, observations were censored in the descriptive analysis and imputed as physiologically normal values for age for the predictive models, as what was done for most ICU severity scores (19–22).

### Predictive Models

We aimed to for an early prediction of which patients will need the longest ventilation time. We followed the TRIPOD (23) and the 2020 ICU predictive model guidelines (24) to build the models. A previous work at our institution showed that the 75th percentile of the total ventilation time (invasive or not) was at 125 days (25). We built four machine learning classifiers to identify the most severe quartile based on the available data at inclusion. We selected four algorithms that can efficiently learn from a small dataset and with few hyperparameters to set: logistic regression with penalization (Elastic Net), Naive Bayes methods, random forests, and eXtreme Gradient Boosting (26). All models were built using Python v3.9 (Python Programming Language, RRID:SCR_008394), scikit-learn v0.24 library (scikit-learn, RRID:SCR_002577), and XGBoost library (27). XGBoost and random forests are both decision tree ensemble algorithms. However, random forests rely on bagging, which is a democratic process to “elect” the best decision among the subgroups of trees (28). XGBoost is based on a boosting process, which is an ensemble of weak learners that is reinforced depending on the quality of the assessment. Both are effective in the medical fields (29). The hyperparameters, if applicable, were optimized to minimize the error reported.

We consulted the PICU and NICU specialists in our institution to establish the list of variables to be included in the models. After an item generation process was completed with data in the literature (1), elements with full agreements were selected to be finally included in the models.

We used a Monte Carlo cross validation method to estimate an empiric distribution from our dataset. This method allows for the validation of our models without an external cohort and estimates of 95% confidence intervals (30). We randomly divided with stratification into a train (70%) and a test (30%) set. We repeated the split processes (bootstraps) for *N*^2^ times (*N* = number of patients) (31) and calculated the discrimination ability of the models on the test cohort with the area under the receiver operating characteristic curve (AUROC), its 95% confidence interval (95% CI), and the *P*-values when comparing different algorithms (32, 33). We determined the importance of each variable using the permutation method (28). Convergence of the best models was assessed using the cumulative mean of the AUROC for each consecutive iteration.

## Results

**Figure 1:**
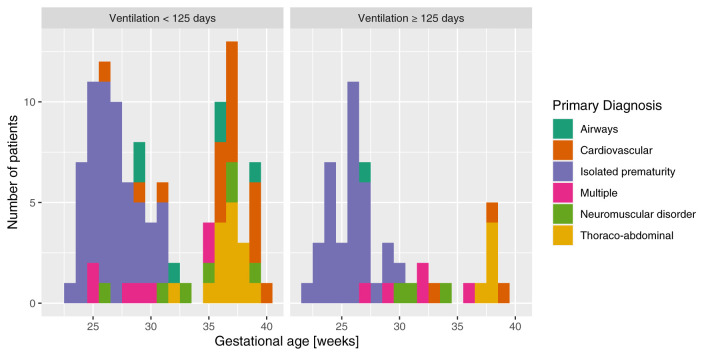
Primary diagnosis categories distributed per gestational age.

During the 10-year period, 9,726 infants were admitted to the NICU and 52% (*n* = 5,087) had at least one ventilatory episode. Of these, 164 met the inclusion criteria after a full review of the charts. No patients were excluded. Forty percent (*n* = 66) of the patients were female. Median gestational age was 29 weeks (IQR: 26–36), with a bimodal distribution (Figure 1 and Supplementary Table 1). Birth weights were approximately normal (median *Z*-score: −0.3, IRQ: −1.3–0.3). Other demographic and ventilation data are presented in Tables 1, 2. Clinical definitions used to define the diagnoses are presented in Supplementary Table 2.

**Table 1:** Patient characteristics.

**Variables**	**All children** **(*n* = 164)**	**Ventilation days** **≥125 days** **(*n* = 52)**	**Ventilation days** **<125 days** **(*n* = 112)**	***P***
	***n*** **(%) or median [IQR]**			
Female, *n*	66 (40)	15 (29)	51 (46)	0.059
Gestational age, week	29 [26–36]	27 [25–31.2]	30 [26–36]	0.005
Gestational age, strata
<28 week	73 (45)	32 (62)	41 (37)	0.004
28 to 32 week	36 (22)	9 (17)	27 (24)	0.42
33 to 36 week	24 (15)	4 (8)	20 (18)	0.10
≥37 week	31 (19)	7 (14)	24 (21)	0.29
Birth Weight, g	1,065 [770–2,155]	810 [690–1,343]	1,205 [820–2,496]	<0.001
Birth Weight, *Z*-score	−0.3 [−1.3–0.3]	−0.6 [−1.3–0]	−0.2 [−1.2–0.4]	0.09
Delivery, cesarean	112 (68)	35 (67)	77 (69)	0.86
Delivery room intubation, *n*	89 (54)	31 (60)	58 (52)	0.40
Apgar scores, strata
1-min Apgar score	4 [2–7]	4 [2–6]	4 [2–7]	0.42
5-min Apgar score	6 [5–8]	6 [5–7.5]	6 [5–8]	0.40
10-min Apgar score	7 [6–9]	7 [6–8.5]	7.5 [6–9]	0.08
Arterial cord pH	7.3 [7.2–7.3]	7.3 [7.2–7.3]	7.3 [7.2–7.3]	0.48
Total ventilation time, days	104 [66–139]	172 [141–236]	84 [51–106]	<0.001
High-frequency oscillatory ventilation, days and *n*	22.8 [10.5–40.5]	35.1 [17.7–45]	18.1 [8.1–35]	0.005
	131 (80)	45 (87)	86 (77)	0.21
Conventional Mechanical Ventilation, days and *n*	23 [12.1–50.8]	37.2 [16.1–116.7]	21.4 [10.5–40.3]	0.007
	161 (98)	51 (98)	110 (98)	>0.99
Noninvasive Ventilation, days and *n*	29.6 [11–52]	52.7 [29.4–88.1]	22.9 [6–40.9]	<0.001
	154 (94)	51 (98)	103 (92)	0.17
High Flow Nasal Cannula, days and *n*	31.6 [14.7–44.7]	48.8 [34.8–98]	20.4 [13.8–32]	<0.001
	75 (46)	31 (60)	44 (39)	0.019

*IQR, Interquartile Range*.

**Table 2:** Characteristics at inclusion.

**Variables^**a**^**	**All children** **(*n* = 164)**	**Ventilation Days** **≥125 days (*n* = 52)**	**Ventilation Days** **<125 days (*n* = 112)**	**Adjusted Odds** **Ratios^**c**^ (95% CI)**	***P***
	***n*** **(%) or median (IQR)**				
Weight at inclusion, *Z*-score	−0.5 [−1–0.3]	−0.8 [−2–0.1]	−0.5 [−1–0.3]	0.67 (0.48–0.89)	0.009
PCO_2_, mmHg	62 [53–68]	67 [61–71]	60 [52–66]	1.02 (0.99–1.06)	0.23
Bicarbonate, mEq/L	31 [28–34]	33 [30–35]	30 [28–33]	1.03 (0.96–1.11)	0.39
Invasive ventilation	61 (37)	18 (35)	43 (38)	1.44 (0.65–3.24)	0.37
PEEP, cmH_2_O	6 [5–6]	6 [5–6]	5 [5–6]	1.31 (0.91–1.94)	0.16
FiO_2_, %	30 [25–40]	40 [30–46]	28 [23–40]	1.01 (0.99–1.03)	0.41
SpO_2_, %^b^	94 [93–96]	94 [92–96]	94 [93–96]	1.03 (0.96–1.11)	0.47
SpO_2_/FiO_2_ ratio	313 [224–384]	248 [191–322]	333 [238–401]	1 (0.99–1)	0.04
Diagnosis^b^					
Pulmonary hypertension	70 (43)	24 (46)	46 (41)	1.55 (0.77–3.18)	0.22
Early pulmonary dysplasia^c^	67 (41)	20 (38)	47 (42)	0.85 (0.42–1.7)	0.64
Lobar emphysema	60 (37)	16 (31)	44 (39)	0.62 (0.3–1.28)	0.20
Patent ductus arteriosus	28 (17)	8 (15)	20 (18)	0.91 (0.34–2.23)	0.84
Cardiovascular disease	26 (16)	6 (12)	20 (18)	1.27 (0.4–3.81)	0.67
CDH or pulmonary hypoplasia	16 (10)	5 (10)	11 (10)	1.98 (0.52–7)	0.29
Neuromuscular disease	13 (8)	3 (6)	10 (9)	0.81 (0.17–2.9)	0.76
Esophageal atresia	10 (6)	3 (6)	7 (6)	1.62 (0.32–6.8)	0.52
Polymalformative syndrome with heart disease	10 (6)	2 (4)	8 (7)	0.92 (0.13–4.2)	0.92
Sepsis	10 (6)	3 (6)	7 (6)	0.75 (0.17–2.44)	0.65
Tracheobronchomalacia	10 (6)	1 (2)	9 (8)	0.31 (0.02–1.82)	0.28
Surgical necrotizing enterocolitis	9 (5)	3 (6)	6 (5)	1.03 (0.2–4.33)	0.97
Polymalformative syndrome with airways lesion	8 (5)	2 (4)	6 (5)	1.07 (0.15–5.27)	0.94

*IQR, Interquartile range; CI, confidence interval. Odds ratio were adjusted for gestational age and sex. CDH, Congenital diaphragmatic hernia; PEEP, positive end-expiratory pressure*.

a *Most recent value before inclusion*.

b *Total is not equal to 100% because each patient can have more than one diagnosis*.

c *Adjusted for gender and gestational age*.

Outcomes at 18 months are presented in Table 3, and showed that 29% (*n* = 47) of the patients had died. Among the survivors, 83% (*n* = 97) were completely weaned of any respiratory or oxygen support. Three patients (2%) were still tracheotomized at this point. Exclusive oral feeding was achieved for 72% of the patients. However, they were slightly underweight with a −0.9 *Z*-score (IQR: −2 to 0). Their neurological functional status was also impacted with a median PCPC and POPC score of 3 (IQR: 2–3), corresponding to “moderate disability” (i.e., special classrooms).

**Table 3:** Outcomes at 18 months corrected age.

**Variables**	**All children** **(*n* = 164)**	**Ventilation days ** **≥125 days (*n* = 52)**	**Ventilation days ** **<125 days (*n* = 112)**	**Adjusted odds ratios^**b**^ (95% CI)**	***P***
	***n*** **(%) or median [IQR]**				
Mortality	47 (29)	11 (21)	36 (32)	0.68 (0.3–1.5)	0.355
Lost–follow-up or inadequate information–assess	8 (5)	1 (2)	7 (6)	0.39 (0.02–2.42)	0.396
Hospitalized at 18 months of corrected age	4 (3)	3 (7)	1 (1)	4.56 (0.53–96)	0.204
**Respiratory status**^a^					
No respiratory support	97 (83)	31 (76)	66 (87)	0.35 (0.12–1)	0.052
Invasive ventilation	2 (2)	1 (2)	1 (1)	4.55 (0.16–127)	0.307
Noninvasive ventilation	3 (3)	3 (7)	0	–	–
High flow nasal cannula	1 (1)	1 (2)	0	–	–
Standard nasal cannula	6 (5)	4 (10)	2 (3)	5.35 (0.89–45)	0.079
Tracheostomy	3 (2)	1 (2)	2 (2)	0.66 (0.03–7.7)	0.745
**Nutritional status**^a^					
Exclusive oral feeding	74 (72)	20 (51)	54 (84)	0.09 (0.02–0.27)	<0.001
Weight, kg	10 [9–11]	10 [9–11]	10 [9–12]	0.75 (0.53–1.02)	0.087
Weight, *Z*-score	−0.9 [−2–0]	−0.9 [−2 to −0.4]	−0.9 [−1–0.7]	0.69 (0.46–1)	0.063
**Neurological status**					
PCPC	3 [2–3]	3 [2–3]	2 [2–3]	2.28 (1.29–4.36)	0.008
POPC	3 [2–3]	3 [2–3]	2 [2–3]	1.96 (1.21–3.33)	0.008

*IQR, Interquartile range; PCPC, Pediatric Cerebral Performance Category; POPC, Pediatric Overall Performance Category*.

a *Only available data were used to calculate percentages*.

b *Adjusted for gender and gestational age*.

### Subgroup of Patients Ventilated for ≥125 Days

Patients ventilated for ≥125 days had a lower female proportion (29%, *n* = 15) than the subgroup ventilated for <125 days (46%, *n* = 51), but it did not reach a statistical significance (*P* = 0.06). The weight *Z*-score at inclusion was significantly lower in the subgroup ≥125 days (aOR: 0.67, 95% CI: 0.48–0.89, *P* = 0.009). However, the ventilatory characteristics and diagnoses were not significantly different when adjusted for gestational age and gender. A comparison of patients' characteristics is shown in Tables 1, 2. Unadjusted odds ratios are presented in Supplementary Tables 3, 4.

There is no significant difference in mortality at 18 months corrected age (Table 3) in the subgroup ventilated for <125 days (32%) compared to those ventilated for ≥125 days (21%, aOR: 0.68, 95% CI: 0.3–1.5). Survivors ventilated for ≥125 days subgroup had a lower proportion of being weaned off any respiratory support (aOR: 0.35, 95% CI: 0.12–1, *P* = 0.06) when adjusted for gestational age and gender. Exclusive oral feeding was higher for patients ventilated for <125 days (aOR: 0.09, 95 CI: 0.02–0.27, *P* <0.001). Neurological impairment was also less severe in the <125 days subgroup (median PCP: 2, IQR: 2–3, and median PCPC: 3, IQR: 2–3 with *P* = 0.008, respectively).

### Evolution of Ventilation Modalities

**Figure 2:**
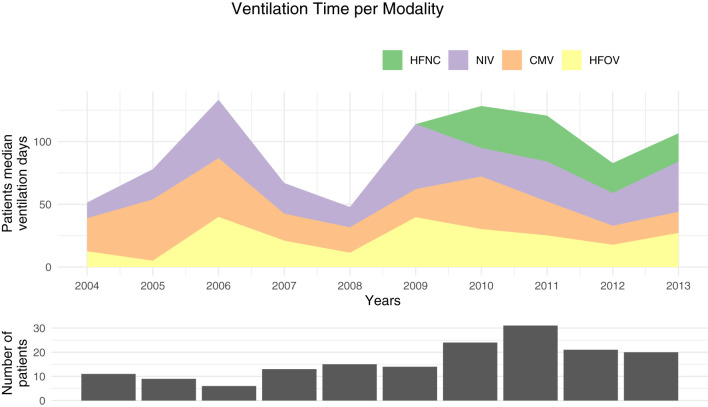
Median ventilation time per patient for each modality. HFNC, high-flow nasal canula; NIV, noninvasive ventilation; CMV, conventional mechanical ventilation; HFOV, high frequency oscillatory ventilation.

**Figure 3:**
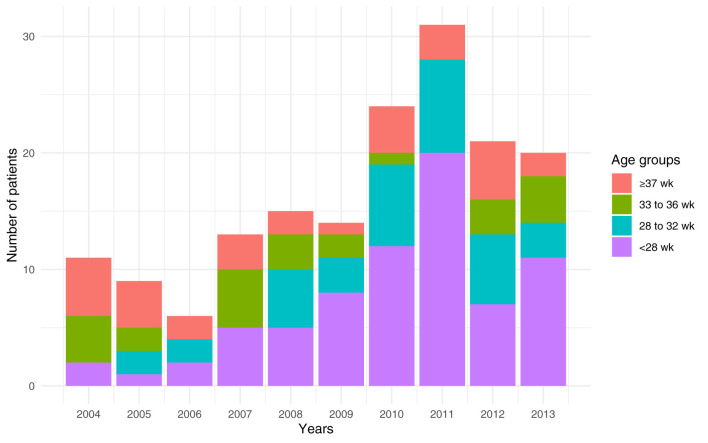
Gestational age distribution per year of inclusion. wk, weeks.

Ventilation modalities evolved over time. Our study covered from 2004 to 2013 and showed the introduction of high-flow nasal cannula (HFNC) in 2010 (Figure 2). We compared the median duration for each modality before (2004–2008) and after HFNC was introduced (2009–2013). The median noninvasive ventilation (NIV) duration was 21 days (IQR: 5–57) before and 34 days (IQR: 16–52) after HFNC implementation (*P* = 0.20). The conventional mechanical ventilation had a median duration of 23 days before 2009 (IQR: 17–79) and 23 days from 2009 to 2013 (IQR: 9–42, *P* = 0.09). The high frequency oscillatory ventilation (HFOV) had a median duration of 15 days before 2009 (IQR: 6–34) and 27 days after (IQR: 12–41 days, *P* = 0.009). The median total ventilation time went from 94 days (IQR: 42–119) to 110 days (IQR: 83–142, *P* = 0.02) after 2009. If HFNC were excluded after 2009, the median time was 85 days (IQR: 63–110) which was not different from the period before 2009 (*P* = 0.79). Furthermore, the number of patients included per year (Figures 2, 3) linearly increased by about two patients per year (*P* = 0.008).

We also showed that the number of preterms born before <28 weeks increased in the observation period (Figure 3).

### Predictive Models

All models included 26 variables (Supplementary Table 2). Among the four tested models, random forests and XGBoost showed the highest discrimination with an AUROC of 0.65 (95% CI: 0.54–0.72, *P* = 0.008) and 0.62 (95% CI 0.50–0.70, *P* = 0.025), respectively. The difference was not statistically significant (*P* = 0.41). Logistic regression with penalization and naive Bayes showed a weak discrimination with an AUROC of 0.58 (95% CI: 0.46–0.66, *P* = 0.09) and AUROC of 0.53 (95% CI: 0.45–0.62, *P* = 0.19), respectively.

**Figure 4:**
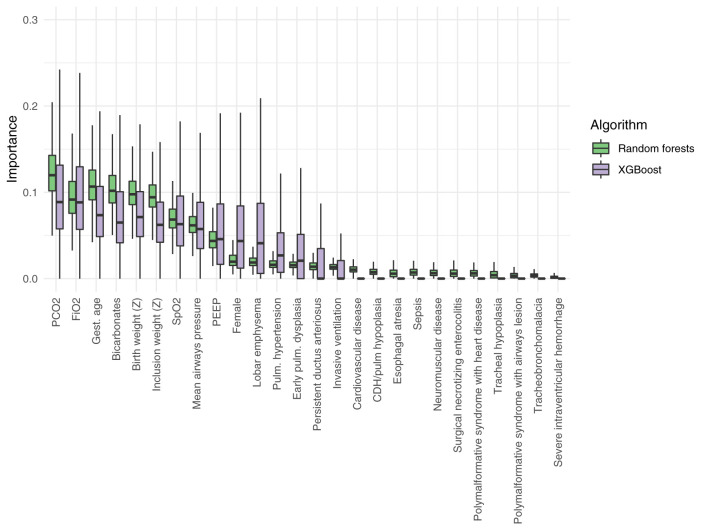
Relative importance of the different variables included in the random forests and in the extreme gradient boosting models (XGBoost). PEEP, positive end expiratory pressure; CDH, congenital diaphragmatic hernia; *Z, Z*-score.

The important analysis (Figure 4) of the random forests and XGBoost models showed that blood gas results close to the inclusion (PCO_2_ and bicarbonate) provided important features for the models, so as the inspired oxygen concentration and gestational age, birth weight, and weight at inclusion *Z*-score. Diagnoses were generally less contributive to the model. The most contributive variables were similar among random forest and XGBoost algorithms.

Convergence of the best models (random forests and XGBoost) was achieved after about 10,000 Monte Carlo repetitions (Supplementary Figure 1). Random forest was optimal with a maximum depth of 300 trees, logistic regression used an L1 ratio of 0.5, and XGBoost used a maximum of 100 boosting rounds. By definition, no hyperparameter optimization was required for the naive Bayes classifier.

## Discussion

We described a diversified cohort of 164 hospitalized newborns who had NPMV with their outcomes at 18 months corrected age. Although this condition is rare, this study indicates that it is a severe one with multiple complex associated comorbidities. This study also suggests that the number of patients and ventilation duration are increasing over the studied period. As previously shown, these children required complex multidisciplinary care and have a significant economic and logistical burden with a high mortality rate. Moreover, most survivors developed a significant neurological impairment, even if most of them are successfully weaned of any respiratory or nutritional support. Several causes can contribute to the neurological impairment in this heterogenous population (for example, intra-ventricular hemorrhage, hypoxic ischemic encephalopathy, genetic susceptibility, low cardiac output, etc.). The problem was already described more than three decades ago, but very few recent studies presented an updated description of the situation, including the increased use of modern ventilation modes, such as high flow nasal cannula (12).

About 80% of the cohort was born prematurely, but with a similar proportion in the subgroup ventilated for <125 and ≥125 days. Once adjusted for gestational age and gender, no diagnoses were shown to be significantly associated with both subgroups. At inclusion, the patients who received ≥125 days of ventilation were significantly smaller (weight *Z*-score) than those who received <125 days of ventilation. Nutrition has been closely linked to respiratory failure in newborns, but the causal relationship is highly complex to establish (34). In our study, birth weight *Z*-scores were similar between groups, suggesting that the difference can be acquired between birth and inclusion. This can be the consequence of the underlying disease severity or the direct cause of the sustained ventilation. This study also showed that our center rarely used tracheostomies in the <125 days subgroup, although many benefits of this intervention, including an improved nutrition and growth, have been suggested (35). Moreover, we found no difference between the groups on the use of invasive ventilation or other ventilator parameters. However, one should note that more than half of the patients in the ≥125 days of total ventilation had a hypoxemia level at inclusion (SF median: 248, IQR: 191–322) comparable to the pediatric acute respiratory distress syndrome which has an SF threshold of 264 (36). Blood gases were not different between the groups at inclusion, and both had high PCO_2_ levels (median PCO_2_: 62, IQR: 53–68).

The outcomes at 18 months corrected age showed a high mortality rate (29%), which is one of the highest mortality rates seen in modern PICUs that usually have an overall mortality below 5% (37). In 1987, a similar study described a mortality of 25% at 2 years of age (12). However, the authors described that 25% of the survivors still required a ventilation support even after 18 years of follow-up. In our study, we observed that 17% of the patients still needed a support at 18 months and most of them were at home with a standard nasal cannula. Only 2% of the patients still required invasive ventilation at this point. At 18 months, patients were generally underweight (median *Z*-score: −0.9) for both groups (*P* = 0.06). Exclusive oral feeding was achieved in 51% (*n* = 20) and 84% (*n* = 54) of the ≥125 and <125 days of ventilation groups, respectively (*P* <0.001). Neurological impairment evaluation is usually difficult and limited in retrospective studies. However, PCPC and POPC scores have been validated for that purpose (18). Overall, the cohort showed a moderate disability (median: 3, IQR: 2–3) which corresponds to “sufficient cerebral function for age-appropriate independent activities of daily life” but children usually require “special education classroom and/or learning deficit present” (18). The disability was less marked in the <125 days of ventilation subgroup (median: 2) compared to the ≥125 days of ventilation subgroup (median: 3, *P* = 0.008). An association between a longer ventilation time and a worse neurological outcome is known for newborns (13, 14). However, the causality is once again complex to determine since multiple confounding variables are present.

Ventilation modalities have evolved in the last decade with an increasing NIV and HFNC as well as the total ventilation time per patient as shown in our data (Figure 2) and in the literature (38). As HFNC was introduced in 2010, one may ask if the increase was only due to this new modality or if this mode replaces another one. Our data showed that the total duration is no longer different if HFNC is removed from the equation and that invasive and NIV median time were similar before and after the HFNC introduction in this population. Although HFNC are known to have a real respiratory support effect (4), without any objective measurement of the lung function to compare these patients, we can only speculate between a lower clinical threshold or an increased respiratory failure severity. Our data also showed that the median time on HFOV nearly doubled before and after 2010. It may be associated with the increase of preterms <28 weeks over the years (Figure 3) as, in our institution, this population was more frequently ventilated with HFOV.

The study has strengths. First, the number of patients is large for the field. Very few studies focused on NPMV for patients that cannot be discharged to a home ventilation program. Because both the number of patients and length of ventilation are increasing, having more data on the topic is essential. Second, we chose to take a pragmatic point of view when describing the diversified cohort of preterm and term neonates (Figure 1). Because these patients are sharing the same resources in the hospital, we argue that they will benefit from a global and comprehensive analysis. Third, this study is the first to our knowledge to publish a predictive model for the early identification of neonates diagnosed with NPMV that need to be ventilated for ≥125 days. Fourth, we used modern algorithms to isolate the most important variables that guided the model to identify the most severe patients. These data will help with the hypothesis generation.

This study has limitations. First, NPMV is a rare entity and the underlying diagnoses even more so. Thus, it is difficult to detect a statistically significant difference between the subgroups of patients ventilated for ≥125 days or less. To overcome this limitation, our center is piloting a prospective cross-sectional multicentric study on the topic (Long VentKid: https://longventkids.ca) with the collaboration of many international pediatric intensive care societies. The second limitation is the retrospective nature of the study, especially for the accuracy of some clinical diagnoses such as pulmonary hypertension, patent ductus arteriosus, or tracheobronchomalacia. As these diagnoses were only assessed if they were clinically relevant, prevalent data may be biased. Furthermore, some diagnoses are subjective, especially those that rely on radiological data. We limited the inter-observer variability by requiring that a minimum of two reports confirmed the diagnosis or to be proven by the gold standard (e.g., tracheobronchomalacia had to be proven by endoscopy). Nevertheless, the only differences between the groups were about the objective data.

## Conclusion

We described the main characteristics and the outcomes at 18 months corrected age of neonates that required a prolonged mechanical ventilation. We showed that the mortality is high, most patients have a significant neurological impairment, and half of those with the longest ventilation duration were exclusively orally fed at 18 months. However, only 2% of the survivors were still requiring invasive ventilation at 18 months corrected age. Future interventional studies should answer if an early and multidisciplinary intensive therapy on the most at-risk patients can improve their outcomes.

## Data Availability Statement

The original contributions presented in the study are included in the article/Supplementary Materials, further inquiries can be directed to the corresponding author/s.

## Ethics Statement

The studies involving human participants were reviewed and approved by Comité d'éthique de la recherche CHU Sainte-Justine, Montréal, Canada. Written informed consent from the participants' legal guardian/next of kin was not required to participate in this study in accordance with the national legislation and the institutional requirements.

## Author Contributions

MS conceptualized and designed the study, collected the data, performed the statistical analysis, and drafted the manuscript. KB collected the data and reviewed the manuscript. PJ and GE conceptualized and designed the study and revised the manuscript. AK reviewed the initial manuscript and gave advices from the statistical and methodological perspectives. GL and NS critically reviewed the manuscript for important intellectual content. All authors approved the final manuscript as submitted and agreed to be accountable for all aspects of the work.

## Conflict of Interest

The authors declare that the research was conducted in the absence of any commercial or financial relationships that could be construed as a potential conflict of interest. The handling editor declared a past co-authorship with one of the authors GE.
