# Supplementary material for: Long-Term Mechanical Ventilation in Neonates: A 10-Year Overview and Predictive Model
Source: Front Pediatr. 2021 Jul 13;9:689190. doi: 10.3389/fped.2021.689190 (PMC8313736; doi:10.3389/fped.2021.689190)
Supplement: Supplementary file 1 [file Data_Sheet_1.pdf]

![frontiers]

Long Term Mechanical Ventilation in Neonates

Sauthier M, Sauthier N, Bergeron Gallant K, Lodygensky G, Kawaguchi A, Emeriaud G, Jouvet P

Supplementary Material

1 Supplementary Tables

1.1 Supplementary Table 1: Number of Patients per Gestational Age and per Primary Diagnosis

**Ventilation**	**Primary Diagnosis**	**Gestational age (weeks)**																		
		22	23	24	25	26	27	28	29	30	31	32	33	34	35	36	37	38	39	40
< 125 days	Airways	0	0	0	0	0	0	0	2	0	0	1	0	0	0	2	0	0	1	0
< 125 days	Cardiovascular	0	0	0	0	1	0	0	1	0	1	0	0	0	0	4	6	0	4	1
< 125 days	Isolated prematurity	0	1	7	9	10	10	5	4	3	4	0	0	0	0	0	0	0	0	0
< 125 days	Multiple	0	0	0	2	0	0	1	1	1	0	0	0	0	2	0	0	0	0	0
< 125 days	Neuromuscular disorder	0	0	0	0	1	0	0	0	0	1	0	1	0	1	0	2	0	1	0
< 125 days	Thoraco-abdominal	0	0	0	0	0	0	0	0	0	0	1	0	0	1	4	5	3	1	0
≥ 125 days	Airways	0	0	0	0	0	1	0	0	0	0	0	0	0	0	0	0	0	0	0
≥ 125 days	Cardiovascular	0	0	0	0	0	0	0	0	0	0	0	1	0	0	0	0	1	1	0
≥ 125 days	Isolated prematurity	1	3	7	3	11	5	1	2	1	0	0	0	0	0	0	0	0	0	0
≥ 125 days	Multiple	0	0	0	0	0	1	0	1	0	0	2	0	0	0	1	0	0	0	0
≥ 125 days	Neuromuscular disorder	0	0	0	0	0	0	0	0	1	1	0	0	1	0	0	0	0	0	0
≥ 125 days	Thoraco-abdominal	0	0	0	0	0	0	0	0	0	0	0	0	0	0	0	1	4	0	0

1.2 Supplementary Table 2: Variables Definitions

Variables	Definition
Bicarbonates	Measures in an arterial. capillary or venous blood sample
Birth weight z-score	Normalized with Olsen's growth curves
Cardiovascular disease	Congenital heart or vessels structural malformation
Congenital diaphragmatic hernia or pulmonary hypoplasia	Pulmonary hypoplasia relied on a volumetric radiological modality.
Early pulmonary dysplasia [c]	Based on the radiological report during the first week of life.
Esophageal atresia	
Female	
FiO_2	Inspired oxygen concentration
Gestational age	Expressed in weeks
Inclusion weight (Z)	Weight z-score at inclusion using World Health Organization growth curves
Invasive ventilation	Ventilation through an infraglottic device
Lobar emphysema	Lobar emphysema on at least two radiological reports before inclusion
Mean airways pressure	Mean airways pressure read by the ventilator
Neuromuscular disease	Neurological (central or peripheral) disease and/or muscular pathology diagnosed by a pediatric neurologist
PCO_2	CO_2 partial pressure in blood gas (arterial. capillary or venous)
PEEP	Positive end-expiratory pressure set on a ventilator (invasive or not)
Persistent ductus arteriosus	Ductus arteriosus that required an interventional or surgical ligation
Polymalformative syndrome with airways lesion	Congenital malformation touching two organs or more. including the airways
Polymalformative syndrome with heart disease	Congenital malformation touching two organs or more. including the heart
Pulmonary hypertension [b]	Defined by at least two echocardiograms with type II or III septum or a valve regurgitation that allowed for an estimation of the pulmonary pressures to be more than half of systemic pressures (39)
Sepsis	clinically diagnosed with a positive culture
Severe intraventricular hemorrhage	Intraventricular hemorrhage grade 3 and 4 (40)
SpO_2	Pulsed oximetry measuring oxygen hemoglobin saturation
Surgical necrotizing enterocolitis	Necrotizing enterocolitis that required a surgical treatment
Tracheal hypoplasia	Diagnosis proven by direct endoscopy
Tracheobronchomalacia	Diagnosis proven by direct endoscopy

1.3 Supplementary Table 3: Characteristics at Inclusion - Unadjusted Odds Ratios

**Variables** [a]	**All children (n=164)**	**Ventilation Days ≥ 125 days (n=52)**	**Ventilation Days < 125 days (n=112)**	**Unadjusted Odds Ratios** [c] **(95% CI)**	***P***
	**n (%) or median (IQR)**				
Weight at inclusion, Z-score	-0.5 [-1 – 0.3]	-0.8 [-2 – 0.1]	-0.5 [-1 – 0.3]	0.86 (0.66 – 1.12)	0.27
PCO_2, mmHg	62 [53 – 68]	67 [61 – 71]	60 [52 – 66]	1.04 (1.01 – 1.07)	0.01
Bicarbonate, mEq/L	31 [28 – 34]	33 [30 – 35]	30 [28 – 33]	1.07 (1 – 1.14)	0.06
Invasive ventilation	61 (37)	18 (35)	43 (38)	0.85 (0.42 – 1.68)	0.64
PEEP, cmH_2O	6 [5 – 6]	6 [5 – 6]	5 [5 – 6]	1.44 (1.04 – 2.09)	0.04
FiO_2, %	30 [25 – 40]	40 [30 – 46]	28 [23 – 40]	1.01 (0.99 – 1.03)	0.16
SpO_2, % [b]	94 [93 – 96]	94 [92 – 96]	94 [93 – 96]	1.01 (0.96 – 1.09)	0.68
SpO_2/FiO_2 ratio	313 [224 – 384]	248 [191 – 322]	333 [238 – 401]	1 (0.99 – 1)	<0.01
**Diagnosis** [b]					
Pulmonary hypertension	70 (43)	24 (46)	46 (41)	1.23 (0.63 – 2.39)	0.54
Early pulmonary dysplasia [c]	67 (41)	20 (38)	47 (42)	0.86 (0.44 – 1.69)	0.67
Lobar emphysema	60 (37)	16 (31)	44 (39)	0.69 (0.33 – 1.37)	0.29
Patent ductus arteriosus	28 (17)	8 (15)	20 (18)	0.84 (0.32 – 1.99)	0.70
Cardiovascular disease	26 (16)	6 (12)	20 (18)	0.6 (0.21 – 1.52)	0.31
CDH or pulmonary hypoplasia	16 (10)	5 (10)	11 (10)	0.98 (0.29 – 2.85)	0.97
Neuromuscular disease	13 (8)	3 (6)	10 (9)	0.62 (0.14 – 2.15)	0.49
Esophageal atresia	10 (6)	3 (6)	7 (6)	0.92 (0.19 – 3.46)	0.90
Polymalformative syndrome with heart disease	10 (6)	2 (4)	8 (7)	0.52 (0.08 – 2.17)	0.42
Sepsis	10 (6)	3 (6)	7 (6)	0.74 (0.17 – 2.35)	0.63
Tracheobronchomalacia	10 (6)	1 (2)	9 (8)	0.22 (0.01 – 1.24)	0.16
Surgical necrotizing enterocolitis	9 (5)	3 (6)	6 (5)	1.08 (0.22 – 4.28)	0.91
Polymalformative syndrome with airways lesion	8 (5)	2 (4)	6 (5)	0.71 (0.1 – 3.19)	0.68

IQR: Interquartile range. CI: confidence interval. Odds ratio were adjusted for gestational age and sex. CDH: Congenital diaphragmatic hernia. PEEP: positive end-expiratory pressure. [a] Most recent value before inclusion. [b] Total is not equal to 100% because each patient can have more than one diagnosis. [c] Adjusted for gender and gestational age.

1.4 Supplementary Table 4: Outcomes at 18 Months of Corrected Age – Unadjusted Odds Ratios

**Variables**	**All children (n=164)**	**Ventilation Days ≥ 125 days (n=52)**	**Ventilation Days < 125 days (n=112)**	**Unadjusted Odds Ratios [b] (95% CI)**	***P***
	**n (%) or median [IQR]**				
Mortality	47 (29)	11 (21)	36 (32)	0.57 (0.25 – 1.2)	0.15
Lost to follow-up or inadequate information to assess	8 (5)	1 (2)	7 (6)	0.29 (0.02 – 1.72)	0.26
Hospitalized at 18 months of corrected age	4 (3)	3 (7)	1 (1)	5.92 (0.73 – 121.91)	0.13
**Respiratory status [a]**					
No respiratory support	97 (83)	31 (76)	66 (87)	0.47 (0.17 – 1.26)	0.13
Invasive ventilation	2 (2)	1 (2)	1 (1)	1.87 (0.07 – 48.26)	0.66
Noninvasive ventilation	3 (3)	3 (7)	0	-	-
High flow nasal cannula	1 (1)	1 (2)	0	-	-
Standard nasal cannula	6 (5)	4 (10)	2 (3)	4 (0.75 – 29.81)	0.12
Tracheostomy	3 (2)	1 (2)	2 (2)	1.08 (0.05 – 11.51)	0.95
**Nutritional status [a]**					
Exclusive oral feeding	74 (72)	20 (51)	54 (84)	0.19 (0.07 – 0.48)	<0.001
Weight. kg	10 [9 – 11]	10 [9 – 11]	10 [9 – 12]	0.84 (0.63 – 1.11)	0.23
Weight. Z-score	-0.9 [-2 – 0]	-0.9 [-2 – -0.4]	-0.9 [-1 – 0.7]	0.77 (0.53 – 1.09)	0.15
**Neurological status**					
PCPC	3 [2 – 3]	3 [2 – 3]	2 [2 – 3]	2.02 (1.19 – 3.63)	0.01
POPC	3 [2 – 3]	3 [2 – 3]	2 [2 – 3]	1.62 (1.04 – 2.59)	0.04

IQR: Interquartile range. PCPC: Pediatric Cerebral Performance Category. POPC: Pediatric Overall Performance Category. [a] Only available data were used to calculate percentages. [b] Adjusted for gender and gestational age.

2 Supplementary Figures

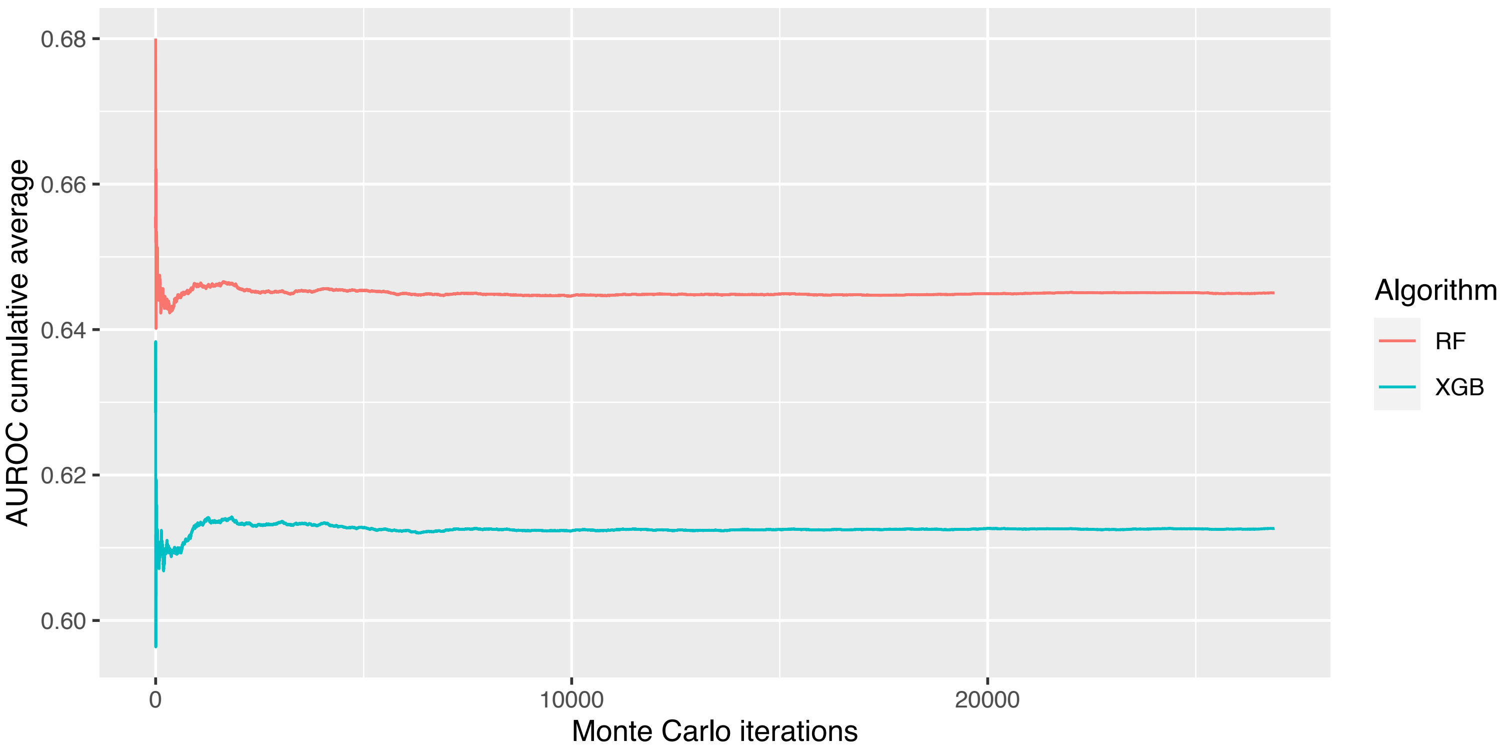

Supplementary Figure 1: Model convergence over the Monte Carlo iterations. RF: random forests. XGB: eXtreme Gradient Boosting
